# Case report: Severe sinus tachycardia as a leading manifestation of systemic lupus erythematosus flare

**DOI:** 10.3389/fmed.2023.1277285

**Published:** 2023-10-13

**Authors:** Anas A. Ashour, Shafik Mansour, Mohamad Talal Basrak, Mohammad Altermanini, Bisher Sawaf, Mohamed A. Atta, Mhd Baraa Habib

**Affiliations:** ^1^Internal Medicine Department, Hamad Medical Corporation, Doha, Qatar; ^2^Emergency Medicine Department, Hamad Medical Corporation, Doha, Qatar

**Keywords:** systemic lupus erythematosus, SLE, flare, arrhythmia, tachycardia, case report

## Abstract

Systemic lupus erythematosus (SLE) is known to have various cardiac manifestations, including arrhythmias and tachycardia. However, it is rare to encounter severe sinus tachycardia as a presenting feature in patients with SLE. Herein, we present a case of a 32-year-old Filipino female with a history of recurrent hospital admissions due to palpitations and uncontrolled tachycardia. Despite trying various symptomatic treatments, her symptoms remained persistent. Ultimately, the patient was diagnosed with SLE during her hospital stay. Eventually, her symptoms improved after initiating steroids. In conclusion, severe sinus tachycardia could be an unusual presentation of SLE that physicians should consider.

## Introduction

Systemic lupus erythematosus (SLE) is a complex autoimmune disorder that can affect multiple organ systems (1). The incidence of cardiac manifestations in SLE patients varies widely, with estimates reaching more than 50% (1). Additionally, the incidence of premature cardiovascular disease in patients with SLE is higher than in the general population (2). Cardiac manifestations are common complications of SLE and can range from mild symptoms, such as palpitations, to life-threatening conditions, such as arrhythmias and myocarditis (1). A wide variety of cardiac arrhythmias have been reported in patients with SLE (3). Such arrhythmias include atrial tachycardia, atrial fibrillation, QTc prolongation, and heart blocks (3). In addition, commonly used medications in treating SLE, such as hydroxychloroquine, can induce QTc prolongation (4). Thus, early diagnosis and treatment of cardiac manifestations are essential in improving the outcomes of SLE patients.

In this report, we describe the case of a young female patient who only experienced recurrent palpitations and severe tachycardia rather than the other typical features of SLE and was hence diagnosed late.

## Case presentation

A 32-year-old Filipino female, hospitalized twice over the last 2 months due to palpitations, came for a third time with progressive palpitations. Her heart rate reached 100–150 beats per minute (bpm). Palpitations were also associated with a generalized skin rash for 3 days. However, the patient denied having fever, chest pain, breathlessness, joint pain, mouth ulcers, or eye pain. She also denied using any new medications. Her medical history is unremarkable, apart from an episode of viral myocarditis diagnosed and treated conservatively 4 months before this presentation. Upon her admission, the cardiac magnetic resonance imaging (MRI) showed mild global hypokinesia with mildly impaired systolic function of 50%, basal inferior wall edema with subepicardial early, and delayed hyperenhancement suggestive of acute myocarditis. As part of investigating the possible causes of myocarditis, autoimmune antibodies were sent before discharge and were to be reviewed in her outpatient appointment.

Furthermore, her previous workup for tachycardia included an ECG, which showed sinus tachycardia with no specific abnormalities, and a follow-up echocardiography and a CT pulmonary angiogram, which were both normal. She only takes bisoprolol 2.5 mg, which was previously prescribed for tachycardia but was ineffective in alleviating her symptoms. She works as an accountant, does not smoke or drink alcohol, and has no significant family history.

**Figure 1:**
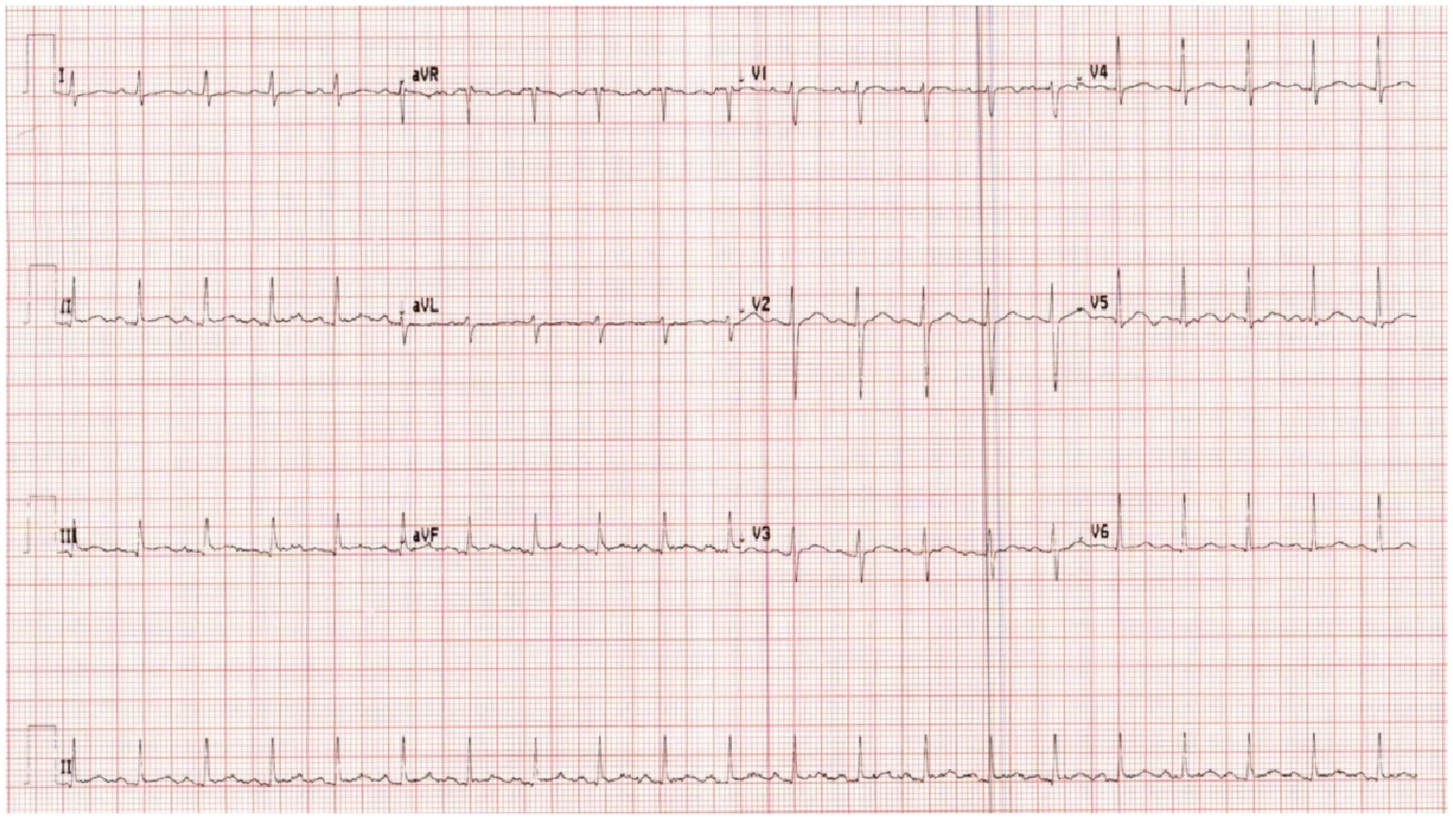
ECG upon admission.

On examination, the patient’s heart rate was 141 bpm, blood pressure was 85/48 mmHg, and multiple erythematous plaques were noted on her face, trunk, and extremities. ECG was done and revealed sinus tachycardia (Figure 1). Laboratory tests showed mild normocytic anemia and moderate proteinuria. However, she had normal values of troponin, C-reactive protein (CRP), procalcitonin, and lactic acid. In addition to a negative viral panel, her urine and blood cultures also did not grow any organism (Table 1).

**Table 1:** Lab values during hospitalization.

Test name	Value w/Units	Normal range
Urine 24-h Protein	1.32 gm/24 h	0.03–0.15
Urea	6.5 mmol/L	2.5–7.8
Sodium	138 mmol/L	133–146
Potassium	4.1 mmol/L	3.5–5.3
Ferritin	2,126.0 μg/L	12.0–160.0
Anti-dsDNA antibodies	>379.00 IU/ml	
Complement C3	0.17 gm/L	0.90–1.80
Complement C4	0.04 gm/L	0.10–0.40
Troponin-T High Sensitivity	8 ng/L	3–10
NT pro-BNP	451 pg/ml	
C-Reactive protein	<2.0 mg/L	0.0–5.0
Procalcitonin	0.62 ng/ml	
White blood cells	5.2 × 10^3/μl	4.0–10.0
Haemoglobin	9.2 gm/dl	12.0–15.0
Lymphocyte count	0.6 × 10^3/μl	1.0–3.0
Creatinine	56 μmol/L	44–80
Adjusted Calcium	2.38 mmol/L	2.20–2.60
Alanine transferase	174 U/L	0–33

The patient was initially treated for possible anaphylaxis with intravenous diphenhydramine and hydrocortisone. Consequently, the rash improved, and the heart rate became normal. After around 8 h, the patient’s heart rate rose again, reaching 150 bpm. The patient was then admitted as a case of possible sepsis and was hence started on piperacillin/tazobactam empirically, although the available data did not suggest any source of infection. The patient also received bisoprolol 2.5 mg, lorazepam 0.5 mg, and intravenous (IV) fluids, but her heart rate remained elevated.

Echocardiography was then performed and showed a normal left ventricular ejection fraction of 54% and no pericardial effusion. Since she was free of chest pain, along with her negative troponin and non-specific ECG findings, cardiac magnetic resonance imaging (MRI) was not indicated and hence not done. CT pulmonary angiogram was negative and ruled out pulmonary embolism. Autoantibodies that were previously sent were noted to be positive with a positive ANA: 1:1280, positive anti dsDNA: >379, low C3: 0.41, low C4: 0.07, in addition to positive anti-RO and anti-Scl-70 (Table 1). Thus, the patient was eventually diagnosed with SLE. Over the next few days, the patient remained tachycardic. Her hospital course was complicated afterward by acute onset psychosis, which was later attributed to SLE neuro-manifestations. However, her brain MRI showed no evidence of cerebritis, encephalitis, or meningitis and no unusual enhancement. A lumbar puncture was also performed and showed a normal cerebrospinal fluid analysis. The patient was then treated with pulse methylprednisolone 500 mg IV daily and hydroxychloroquine 200 mg daily. Afterward, her heart rate gradually decreased from 150 to 95 bpm over a few days, and her psychotic symptoms gradually resolved.

A kidney biopsy was performed in view of proteinuria, which showed focal lupus nephritis, ISN/RPS Class III (activity index 7/24 and chronicity index 1/12). After her clinical status improved, the patient was later discharged on mycophenolate, hydroxychloroquine, and prednisolone 20 mg. A follow-up after 2 months revealed a normal heart rate of around 83 bpm.

## Discussion

This report presents the case of an adult female patient who had frequent healthcare visits for tachycardia with no precise diagnosis. Previous workup of cardiac conditions, including ECG and echocardiogram, revealed no abnormalities. While symptomatic treatment with a beta blocker was initiated, her clinical condition did not improve because treatment of the underlying condition was not initiated. The patient required a medical intensive care unit (ICU) admission on her latest visit due to her tachycardia-induced hypotension. Once the appropriate treatment of SLE was initiated, a gradual response was observed, and the patient’s clinical condition improved. The presented case highlights the diagnostic challenge in a patient with SLE who presented with recurrent tachycardia without any other typical clinical features of the disease. This has led to a delay in the diagnosis and treatment of SLE. Cardiac involvement in the form of arrhythmias and conduction abnormalities is a well-recognized feature of SLE. In a single-center study involving 235 patients with SLE, sinus tachycardia was observed in 18% of the patients, QT prolongation in 17%, and atrial fibrillation in 9% (3). Bradyarrhythmias, including various degrees of heart block, have also been reported to be associated with SLE (5). In contrast to being a well-recognized feature, tachycardia is not a common presenting symptom of SLE and is usually attributed to the primary cardiac involvement in the disease (6).

There have been several reported cases of SLE presenting with tachycardia. For example, Utset et al. (7) suggested sinus tachycardia might reflect the disease activity, with worsening of tachycardia during active flares and resolution of symptoms with treatment of the flare. Such correlation was also noted in our case of this patient, who only controlled her heart rate after managing the active disease (7). Also, Subhani et al. (8) reported a case of a 42-year-old female with SLE who developed focal atrial tachycardia but was incorrectly diagnosed with sinus tachycardia due to an abnormal heart rhythm. Despite receiving antiarrhythmic medications, the patient’s condition worsened. She developed tachycardia-induced cardiomyopathy. Eventually, radiofrequency catheter ablation was performed, leading to a successful recovery of the patient’s heart function (8). In another case, a 17-year-old boy initially presented with supraventricular tachycardia. However, the patient was later found to have pancreatitis, myocarditis, and nephritis, all attributed to the underlying SLE disease. The patient’s echocardiogram also showed left-sided heart failure due to nonischemic cardiomyopathy (9).

Cardiac involvement in SLE is not limited to arrhythmias. Santacruz et al. (10) reported a case of a 40-year-old female who presented with tachycardia, acute dyspnea, and atypical chest pain. ECG was only significant for sinus tachycardia and non-specific T wave changes. The patient was admitted as a case of acute heart failure. However, she rapidly deteriorated, requiring ICU admission. Her hospital course included acute renal impairment, proteinuria, left atrial thrombus, and peri-myocarditis diagnosis. Further workup led to a diagnosis of SLE, which was thought to be the culprit (10).

Another reported presentation was a case described by Zhang et al. (11) of a patient who was admitted with dyspnea, tachycardia, and tachypnea. The patient was found to have cardiac tamponade. Further past medical history revealed joint pain, which, along with positive antibodies, confirmed the diagnosis of SLE. The patient’s condition improved after treating SLE with prednisone and cyclophosphamide (11).

The mechanism by which SLE can cause tachycardia is not fully understood, but it is likely related to the effects of autoantibodies on the cardiac conduction system (12). Autoantibodies such as anti-Ro and anti-La have been implicated in developing conduction abnormalities in SLE patients (12). In addition, SLE-related inflammation and fibrosis may also contribute to tachycardia by inducing oxygen radicals that eventually lead to cardiomyocyte necrosis (13). Furthermore, SLE can cause autonomic dysfunction due to central nervous system involvement or hypothalamic–pituitary–adrenal axis involvement, leading to tachycardia (14).

In the presented case, various differential diagnoses were thought to have caused the patient’s tachycardia before a diagnosis of SLE was established. Such differential diagnoses included anaphylaxis and sepsis. In this case, the patient was treated with pulse methylprednisolone and hydroxychloroquine, which significantly improved her heart rate. This supports the idea that SLE-related inflammation may have contributed to tachycardia in this patient. This case illustrates the importance of awareness of unusual and atypical presentations of systemic autoimmune conditions such as SLE, especially in young females.

## Conclusion

Although sinus tachycardia is one of the common cardiac manifestations of SLE flare, recurrent and severe sinus tachycardia is unusual during the initial presentation without the other classical features of SLE. Physicians should consider SLE flare in patients with sinus tachycardia without a clear explanation. Early recognition and early treatment of cardiac involvement in SLE are crucial to improving patient outcomes.

## Data availability statement

The original contributions presented in the study are included in the article/supplementary material, further inquiries can be directed to the corresponding author.

## Ethics statement

Written informed consent was obtained from the individual(s) for the publication of any potentially identifiable images or data included in this article.

## Author contributions

AA: Visualization, Writing – original draft, Writing – review & editing. SM: Conceptualization, Methodology, Writing – review & editing. MT: Methodology, Writing – review & editing. MAl: Investigation, Writing – review & editing. BS: Writing – review & editing. MAt: Writing – review & editing. MH: Writing – original draft, Writing – review & editing.
